# Blood Biomarkers of Recovery Efficiency in Soccer Players

**DOI:** 10.3390/ijerph16183279

**Published:** 2019-09-06

**Authors:** Anna Nowakowska, Dorota Kostrzewa-Nowak, Rafał Buryta, Robert Nowak

**Affiliations:** Centre for Human Structural and Functional Research, Faculty of Physical Education and Health Promotion, University of Szczecin, 17C Narutowicza St., 70-240 Szczecin, Poland

**Keywords:** exercise, physical fitness, recovery, biochemical markers

## Abstract

Physical exercise strongly affects human metabolism and causes biochemical changes. This study aimed to investigate the relationship between routine plasma biomarker levels and recovery efficiency in soccer players during an entire competitive match season. The players participating in the study were divided into a midfielder/defender group (seven midfielders and seven defenders) and a goalie/substitute group (six persons—goalkeepers and players with a short cumulative match-time). The fasting capillary blood samples were taken 17–24 h after each competitive match. The blood plasma was used to determine the creatinine, urea, alkaline phosphatase, creatine kinase, lactate dehydrogenase, aspartate and alanine aminotransferase, iron and magnesium levels of the athletes. The levels of (AST) (aspartate aminotransferase), (ALT) (alanine aminotransferase) and (Cr) creatinine were higher in the midfielder/defender group than in the control group, but only AST and Cr significantly varied over time (AST decreased, and Cr increased with time). The (LDH) (lactate dehydrogenase) activity and urea level were significantly lower in the midfielder/defender group than in the goalie/substitute group, and it significantly varied over time (LDH decreased, and urea increased with time). No differences in the (CK) creatine kinase and (ALP) alkaline phosphatase activities between the groups was found, although CK increased significantly with time in the midfielder/defender group (particularly midfielders in the spring round). In midfielders, the AST activity and the iron level were significantly lower in the spring than in the autumn round. On the contrary, ALT, CK, urea and magnesium levels were significantly higher in the spring than in autumn round. A long-term measurement of biochemical parameters in elite soccer players indicated that AST, CK, LDH and creatinine levels, when analyzed together, could constitute a useful set of markers for monitoring recovery periods.

## 1. Introduction

Physical exercise strongly affects human metabolism and causes biochemical changes. This is reflected in the results of laboratory analyses of changes in serum parameters, which indicate degrees of fatigue. It is extremely important to provide sport physicians, coaches and all persons involved in sport more comprehensive information about the response of organisms to ordered training [1]. The many faces of different types of training and the variety of physiological reactions of trainees explain why sport medicine is still looking for a perfect way to monitor the effects of training.

There is an enormous need for markers that describe an organisms’ answers to physical exercise. Current knowledge on clinical chemistry is still expanding and growing. There is a continuing search for new markers and parameters that could be useful for daily use. The monitoring of athletes’ training should be performed using measurements of their performance and biological or physiological parameters.

Muscle metabolism parameters, like creatine kinase (CK), lactate dehydrogenase (LDH) and myoglobin, are usually increased after exercise [2]. An increase in these variables may represent an index of cellular necrosis and tissue damage, following acute and chronic muscle injuries [3,4]. Noakes reported that the increase in CK, after long-duration exercise, is more closely related to the duration of exercise than its intensity [5]. Professional soccer players participating in daily training have consistently high resting CK values [6]. The post-exercise growth pattern in CK activity is very specific. CK usually increases after 24–36 h of strenuous exercise, which makes this biomarker a valuable tool for monitoring recovery efficiency. Similarly, LDH, as a muscle damage marker, should be verified [3,7].

Serum creatinine concentration is a known parameter for measuring renal function in clinical medicine. In sports medicine, creatinine is useful as an indicator of the general health status and water-electrolyte balance in a patient. However, it is important to note that serum creatinine concentration is higher in athletes than in the population of people who are not physically active [8]. There is also a strong correlation between the BMI (Body Mass Index) unit, sport performed and creatinine concentration, because there were different kinds of training and involvement of aerobic and anaerobic metabolism [7]. These changes suggest that monitoring the plasma creatinine level should be verified as a recovery efficiency biomarker.

While urea is not specific enough to monitor renal function, monitoring urea concentration in professional athletes can provide some important information. The normalization of the serum urea level might be a signal that a strenuous training session has begun, when this parameter is regularly monitored [9,10,11]. For that reason, the plasma urea level should be considered as a recovery parameter, like creatinine.

Alanine aminotransferase (ALT) and aspartate aminotransferase (AST) serve as markers of liver disease, and increases in AST, ALT, and LDH have been observed, after an endurance effort, such as an ultramarathon [12,13,14]. It was also found that AST, ALT, and γ-glutamyltransferase (GGT) activities could be valuable tools for assessing people’s metabolic response to exercise. Soccer training and matches also induce alkaline phosphatase (ALP), which leaks from the skeletal muscles. ALP is involved in ATP metabolism during exercise by the participation in amino acid catabolism and the hydrolysis of fats across cell membranes [15]. Therefore, monitoring those well-known diagnostic markers, such as enzymes, as mentioned above, could prevent athletes from overreaching or overtraining [16]. Thus, they also could be suitable for recovery efficiency monitoring.

An insufficient recovery time during the soccer match season may lead to fatigue and may predispose players to mineral deficiency. One of the most important micronutrients is iron (Fe), and an iron deficiency may be linked with the development of anemia and the impairment of athletic performance [17,18]. Another relevant micronutrient is magnesium (Mg), which is the cofactor of over 325 enzymatic reactions linked with anaerobic performance, including muscle contraction and relaxation. Mg takes part in regulating troponin expression via calcium ion concentration gradients, which may have an impact on enhancing muscle strength [19,20]. For that reason, measuring plasma iron and magnesium levels in soccer players might be an additional tool for monitoring recovery efficiency.

An ideal biochemical indicator for assessing the degree of body recovery would be a marker that responds only to physical exercise and reacts in a predictable and permanent manner. Every sport has its own specificity. A previous study indicates that elite soccer players are characterized by significant changes in biochemical and hematological parameters over a half-season, which are linked to the training workload, as well as the adaptation induced by the soccer training [21].

No study has yet followed the same team of highly trained professional soccer players repeatedly over a whole season. The present work described the results of a long-term study, performed on elite soccer players of the soccer club, Pogoń S.A. The research was carried out during the whole year of premier league games, both in the spring and autumn rounds.

The aim of the study was to investigate the relationship between routine plasma biomarker levels and recovery efficiency in soccer players during an entire competitive match season.

## 2. Materials and Methods

### 2.1. Study Design

Twenty male professional soccer players of the prime soccer team, MKS Pogoń Szczecin S.A., participated in the study. The total competitive minutes of each player were registered across a whole season, from July until May of the following year (eleven months). A summary of the match-time for each week during the entire period of observation and the cumulative match-time is presented in Table 1. The time spent on the field and the position on the field were differentiating factors in the study. Throughout an entire competitive season, 44 league and cup matches (23 for the autumn round and 21 for the spring round) took place. The matches were usually played each week, except during breaks for matches played by the Polish national soccer team, which sometimes caused an accumulation of league matches (two matches per week). In this period, fasting capillary blood samples were taken 17–24 h after each competitive match in the recovery period, when all players did not perform any intense tasks. The time of blood draws was a compromise which was discussed with the authorities of the club and coaches. The 24th h, after a match, was the only time of rest for the players of this club. There was no other possible time for blood draws during the recovery period. The post-match recovery in soccer players takes usually 48–72 h [22], although many muscle-related parameters have been normalized 24th h after the match [23]. Moreover, Carling et al. have observed in their study, that soccer players usually maintain physical and skill-related performance throughout the season, even when three consecutive games within one week were played [24]. The blood draws at the 24th h after a match were also appropriate due to the CK post-exercise growth pattern (CK activity usually increases after 24 h of strenuous exercise [3,7]). This study’s priority was to examine the real-life situation, since it offered more valuable information for the club than data provided under laboratory conditions. The 7-h range for the blood draws was due to the fact that they took place at different times of the day, when the matches were played.

### 2.2. Participants

The soccer players who participated in the study belonged into three groups: Midfielders (seven players); defenders (seven players); and goalies/substitutes (six players). The goalie/substitute subgroup included three goalies and three players with a short game time per match (study participants, who played for less than 500 min during the entire season). Some of the main characteristics of the study participants (age, height, weight, BMI, length of training experience, number of weekly training h and cumulative match-time) are presented in Table 2. No statistically significant differences in the baseline characteristics were found between these three subgroups, except for the players’ match-time for the entire season, which was significantly lower in the goalie/substitute subgroup in comparison to the midfielders (*p* = 0.05, data not shown in Table 2). Unfortunately, the body composition of the soccer players was not measured, because of a lack of formal consent from the club authorities (additional time for the measurements might disturb the fluency of the training process). This was one of the limitations of our study.

In order to emphasize the impact of the covered distances and metabolic load during a match on the analyzed plasma biomarkers, the study participants were also divided into two subgroups, according to their active play time on the pitch or the differences in the activity levels specific to their position on the field. The first group was composed of midfielders and defenders (fourteen players), and the second group, goalies/substitutes, consisted of goalies and players with a short game time per match (six players). The reason for such division is the fact that players of different positions vary in the energetic and physiological demands due to the differences in their body stature, the distances covered and the activities performed. For example, wide midfielders, as the most active on the field, cover longer distances, perform more sprints, accelerations, and decelerations. Central defenders cover shorter distances and use less metabolic power [25,26,27]. In turn, goalkeepers may have similar or reduced performance in comparison with other field players but are predisposed to higher vertical jumps [28]. All of these factors affect the blood biomarkers related to physical activity [29].

The athletes were non-smokers and had no history of metabolic syndrome or cardiovascular diseases. They took no medication or supplements, which may have impact on their body metabolism. The training plan of the players was relevant to their position on the field. The right and left midfielders and defenders performed interval speed-endurance training. In the central midfielder and defender subgroups, the elements of deceleration, reacceleration and change of direction were added.The goalkeepers performed strength-agility training.

The players were fully informed in advance of any risks and discomfort associated with the blood sampling procedures before giving their written consent to participate. The study was approved by the Local Ethics Committee (No 13/KB/V/2014), in accordance with the Helsinki Declaration.

### 2.3. Blood Sampling

The capillary blood from the fingertip was collected after an overnight fast, according to the standard diagnostic procedures. A capillary blood collection system for biochemical analyses, with a lithium heparin anticoagulant (3 capillaries per 200 μL of blood), and for hematological analyses, with an ethylenediaminetetraacetic acid (EDTA) anticoagulant (one capillary per 200 μL of blood, data not presented), were used to collect the fingertip blood samples. The initial drop of blood was always discarded before collection. The plasma was separated from the blood within a period of up to 2 h after collection and then analyzed.

### 2.4. Biochemical Analyses

The biochemical tests were carried out using an Auto Chemistry Analyzer BM-100 (BioMaxima S.A., Lublin, Poland) in the case of clinical chemistry variables, or an Ion Selective Analyzer BM ISE (BioMaxima S.A., Lublin, Poland) in the case of selected ions. The blood plasma was used to determine the metabolites, creatinine (Cr), urea (U), alkaline phosphatase (ALP), creatine kinase (CK), lactate dehydrogenase (LDH)), aspartate aminotransferase (AST), alanine aminotransferase (ALT), iron (Fe) and magnesium (Mg). All of the studied parameters were determined using a diagnostic method, following the manufacturer’s instructions (BioMaxima S.A., Lublin, Poland). All the analyses were verified using a multiparametric control serum, as well as a control serum of a normal level (BioNorm) and a high level (BioPath) (BioMaxima S.A., Lublin, Poland).

### 2.5. Statistical Analyses

The statistical analyses were carried out using the STATISTICA v 13.1 software statistical package (StatSoft, Inc., 2016), and the statistical significance was set at *p* ≤ 0.05. To test the normal distribution within the subgroups, the Shapiro-Wilk test was used. Asno one fromthe subgroups had a normal distribution of the tested variables, nonparametric statistics were carried out. The results were presented as the medians and interquartile ranges (Q1–Q3). The groups were compared in terms of the plasma levels of the analyzed biochemical parameters using a Mann-Whitney U test. The statistical Friedman’s repeated measures analysis of variance by ranks, with Bonferroni-Dunn’s correction, was used to estimate the significance levels of the observed differences between the analyzed time points during the whole sporting season. The strength and direction of the association between the cumulative match-time and biochemical variables were tested using the nonparametric Spearman’s rank-order correlation.

## 3. Results

### 3.1. Anthropometric Parameters

Table 2 presents some anthropometric characteristics of the participants of the study. No statistically significant differences were found between the subgroups, when considering the age, height, weight, BMI, length of training experience and the number of weekly training hours, except for the match-time for the entire season, which was significantly lower in the goalie/substitute subgroup in comparison to that of the midfielders (*p* = 0.05, data not shown in Table 2).

### 3.2. Comparison of Groups in Terms of Selected Blood Biomarkers

A Mann-Whitney U test was used to compare the studied groups in terms of biochemical variables. Table 3 provides a comparison of the subgroup of midfielders and defenders and the subgroup of goalies/substitutes in terms of biochemical parameters. There was a significant difference between the midfielder/defender and the goalie/substitute subgroups in terms of their AST, ALT and creatinine levels. The activities of both aminotransferases and creatinine levels were higher in the midfielder/defender group than in the goalie/substitute group for the entire season and during the spring match round. In turn, the plasma iron level was significantly lower in the midfielder/defender group than in the goalie/substitute group (entire season and spring round). By contrast, the lactate dehydrogenase activity and urea level were significantly lower in the midfielder/defender group than in the goalie/substitute group.

Table 4 presents the results of the same analyses, comparing the midfielder and defender subgroups. The midfielder group reported a significantly higher activity of both aminotransferases than the defenders for the whole season and the spring round. In turn, the plasma urea level was significantly lower in this group in the autumn round.

Table 5 provides a comparison of the studied subgroups in terms of selected blood biomarkers during the autumn and spring match rounds. In the midfielders, the activity of AST and the iron levels were significantly lower in the spring round than in the autumn round. On the contrary, the ALT, CK, urea and magnesium levels were significantly higher in the spring match round than in the autumn round. In the defenders, significantly lower activities of both aminotransferases and the iron level were seen. In the goalie/substitute group, only a difference in the AST activity was noted. AST was lower in the spring round than in the autumn round.

### 3.3. Variance of Selected Blood Biomarkers in Time

**Figure 1:**
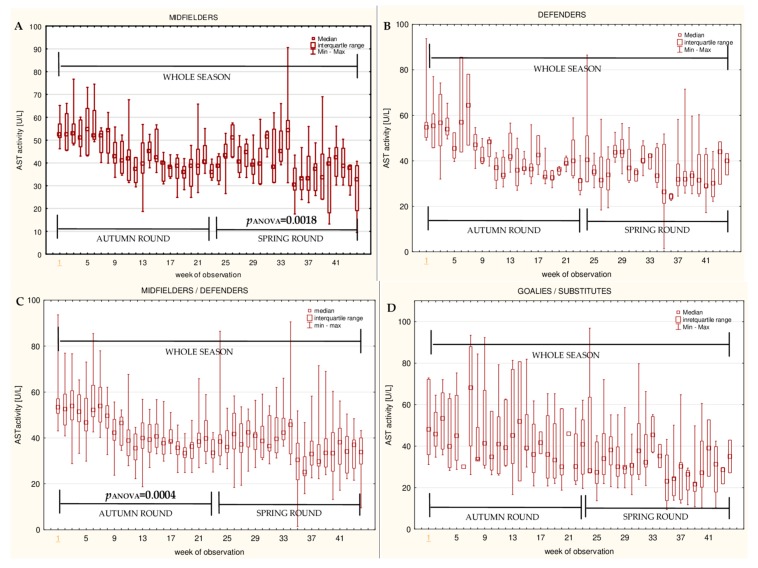
Median aspartate aminotransferase activities (AST) in: (**A**) The midfielder, (**B**) defender, (**C**) midfielder/defender and (**D**) goalie/substitute subgroups over the course of the soccer match season.

**Figure 2:**
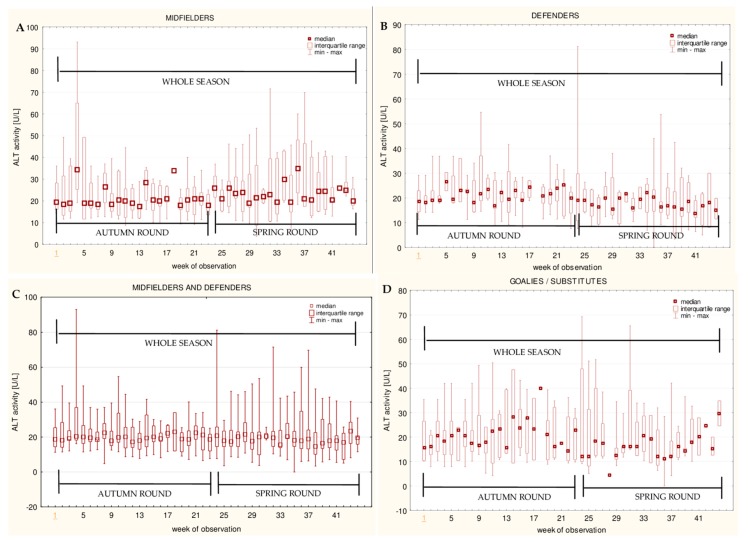
Median alanine aminotransferase (ALT) activities in: (**A**) The midfielder, (**B**) defender, (**C**) midfielder/defender and (**D**) goalie/substitute subgroups over the course of the soccer match season.

**Figure 3:**
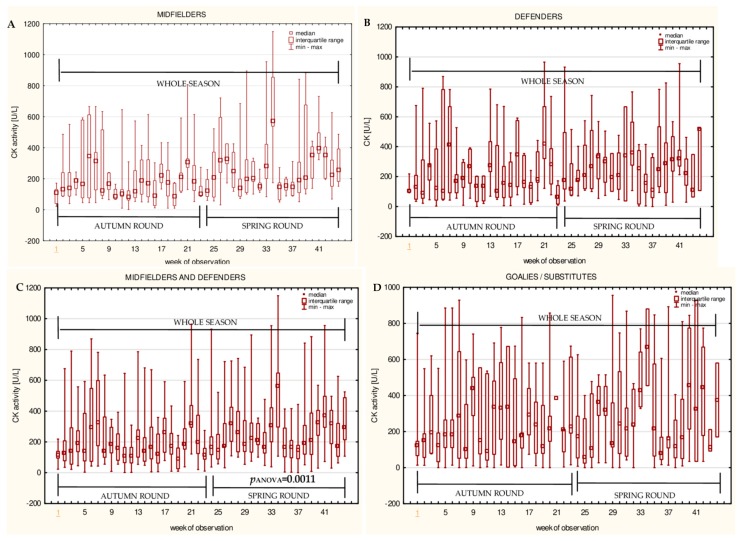
Median creatine kinase (CK) activities in: (**A**) The midfielder, (**B**) defender, (**C**) midfielder/defender and (**D**) goalie/substitute subgroups over the course of the soccer match season.

**Figure 4:**
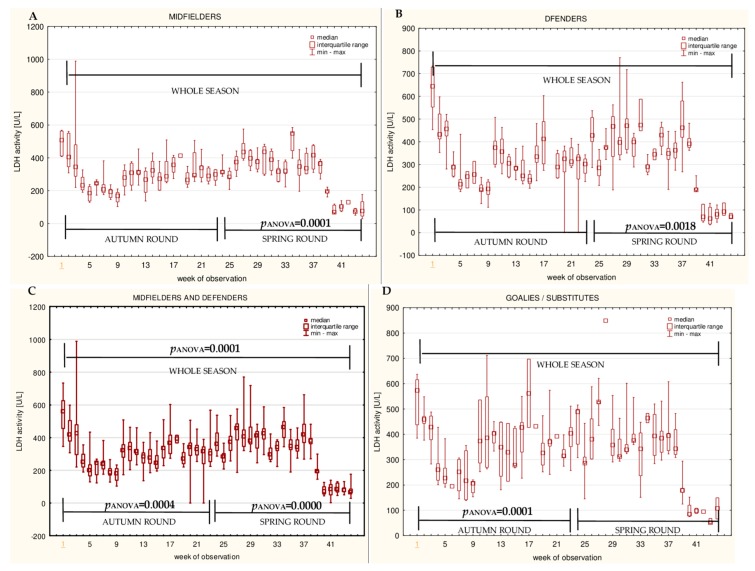
Median lactate dehydrogenase (LDH) activities in: (**A**) The midfielder, (**B**) defender, (**C**) midfielder/defender and (**D**) goalie/substitute subgroups over the course of the soccer match season.

**Figure 5:**
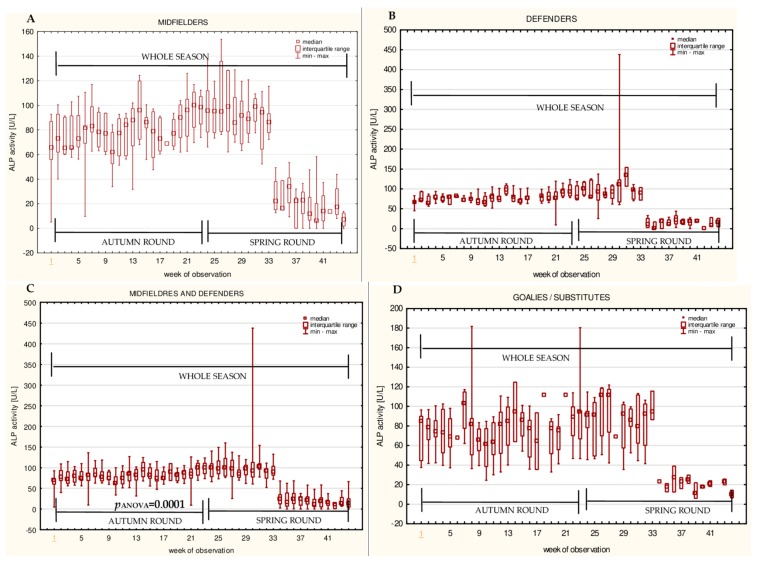
Median alkaline phosphatase(ALP) activities in: (**A**) The midfielder, (**B**) defender, (**C**) midfielder/defender and (**D**) goalie/substitutesubgroups over the course of the soccer match season.

**Figure 6:**
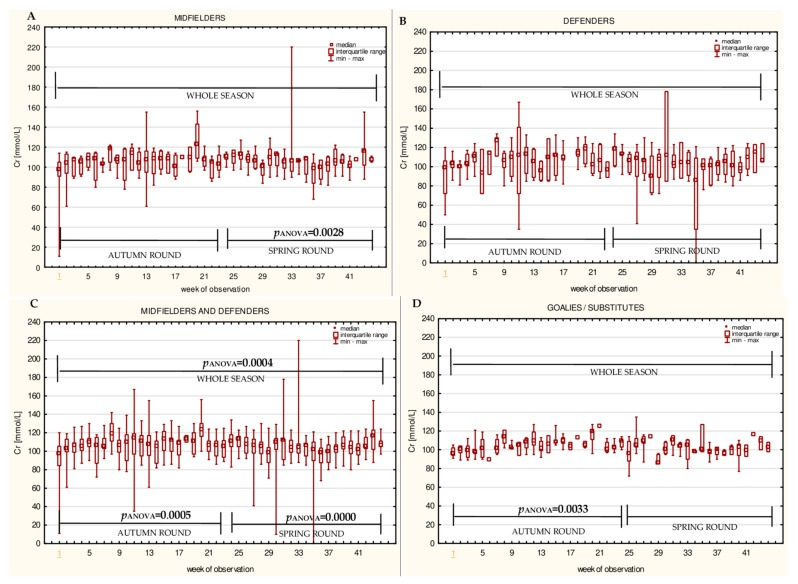
Median creatinine (Cr) levels in: (**A**) The midfielder, (**B**) defender, (**C**) midfielder/defender and (**D**) goalie/substitute subgroups over the course of the soccer match season.

**Figure 7:**
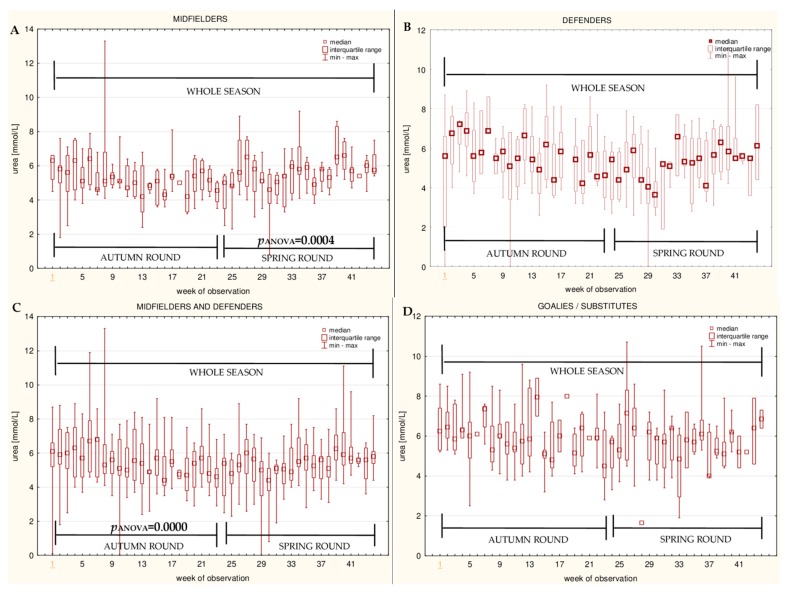
Median urea (U) levels in: (**A**) The midfielder, (**B**) defender, (**C**) midfielder/defender and (**D**) goalie/substitute subgroups over the course of the soccer match season.

**Figure 8:**
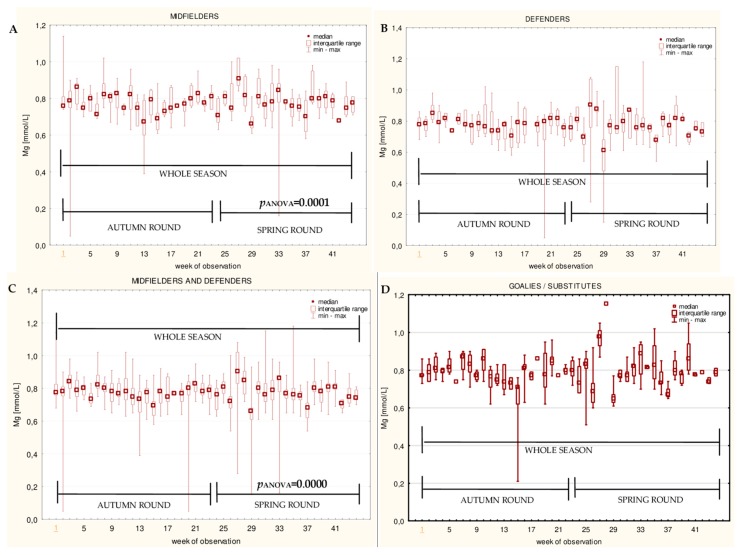
Median magnesium (Mg) levels in: (**A**) The midfielder, (**B**) defender, (**C**) midfielder/defender and (**D**) goalie/substitute subgroups over the course of the soccer match season.

**Figure 9:**
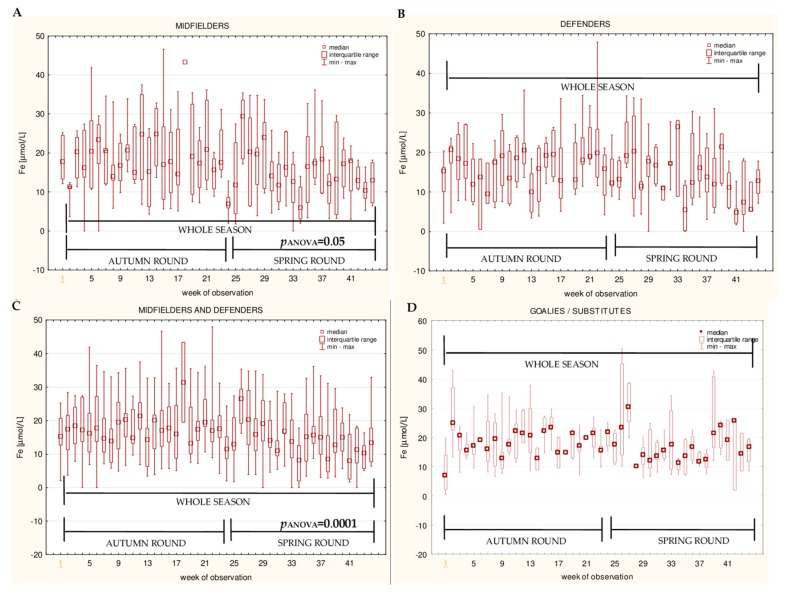
Median iron (Fe) levels in: (**A**) The midfielder, (**B**) defender, (**C**) midfielder/defender and (**D**) goalie/substitute subgroups over the course of the soccer match season.

The data for the longitudinal observation of the median levels of the selected blood biomarkers during the soccer match season, assessed using Friedman’s repeated measures analysis of variance on the midfielder, defender, midfielder/defender and goalie/substitute groups, are presented in Figure 1, Figure 2, Figure 3, Figure 4, Figure 5, Figure 6, Figure 7, Figure 8 and Figure 9. In the midfielder/defender group, the lactate dehydrogenase activity decreased (Figure 4C), the creatinine level varied (Figure 6C), and the magnesium level decreased (Figure 8C) significantly with time, regardless of the phase of the competition. In turn, the creatine kinase activity increased (Figure 3C), the urea level increased (Figure 7C), and the iron level increased (Figure 9C) significantly with time only in the spring round. On the other hand, the aspartate aminotransferase activity significantly decreased (Figure 1C) in the midfielder/defender group only in the autumn round. In the goalie/substitute group, the LDH activity significantly decreased (Figure 4D), and the Cr level increased (Figure 6D) only in the autumn round.

When the midfielders and defenders were analyzed as separate groups, a nonparametric analysis of variance showed a significant effect, mainly in the midfielders, during the spring round, where a variation of the most analyzed parameters was observed. The AST activity decreased (Figure 1A), the LDH activity decreased (Figure 4A), the Cr level increased (Figure 6A), the urea level increased (Figure 7A), and the magnesium level increased (Figure 8A), and iron level decreased (Figure 9A) significantly with time. In the defender subgroup, significant results were noted in the case of the LDH activity (Figure 4B), which also decreased in the spring round.

### 3.4. Correlations between Cumulative Competitive Match-Time and Biochemical Variables

**Figure 10:**
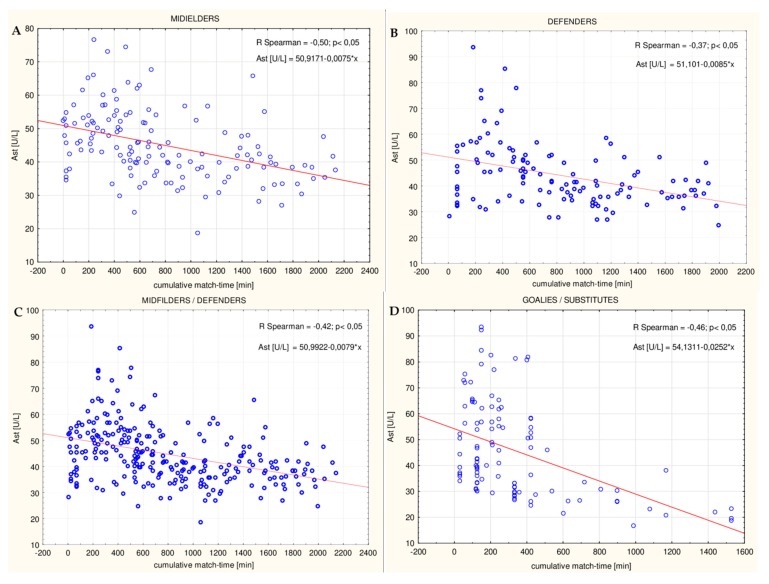
Correlation between cumulative match-time and aspartate aminotransferase activity (AST) in: (**A**) The midfielder, (**B**) defender, (**C**) midfielder and defender, and (**D**) goalie and substitute subgroups over the course of the autumn round of the soccer match season (the Spearman R correlation coefficient was determined).

In the midfielder/defender group, the AST activity was significantly negatively correlated with the cumulative game time for the whole soccer match season (R = −0.42; *p* ≤ 0.05 (Figure 10C), R = −0.33; *p* ≤ 0.05, and R = −0.43; *p* ≤ 0.05 for the autumn round, spring round and entire match season, respectively). A slight significant decrease with time in the LDH and ALP activity during the spring round was also observed (R = −0.29; *p* ≤ 0.05, and R = −0.25; *p* ≤ 0.05 for LDH and ALP, respectively). A slight significant decrease with time in the magnesium level in both rounds (R = −0.16; *p* ≤ 0.05, R = −0.30; *p* ≤ 0.05, and R = −0.17; *p* ≤ 0.05 for the autumn round, spring round and entire match season, respectively) and the iron level in the total season (R = −0.15; *p* ≤ 0.05) was also observed. In the midfielder/defender group, the creatine kinanase activity and creatinine concentration slightly, but significantly, increased with time (R = 0.14; *p* ≤ 0.05 in the autumn round, and R = 0.17; *p* ≤ 0.05 in the total season for CK; and R = 0.25; *p* ≤ 0.05, and R = 0.24; *p* ≤ 0.05 in the autumn and spring round, respectively). A positive correlation between the urea level and the cumulative game time in the spring round was also noted (R = 0.16; *p* ≤ 0.05). An increase in the ALP activity with time in the autumn round and a subsequent decrease in the spring round in the study group was also observed (R = 0.20; *p* ≤ 0.05, and R = −0.25; *p* ≤ 0.05 for the autumn and spring round, respectively). Notably, what is interesting in this datum is that in the goalie/substitute group, the majority of the analyzed biochemical parameters decreased with time. This concerns the activities of AST (R = −0.46 (Figure 10D); *p* ≤ 0.05, R = −0.41; *p* ≤ 0.05, and R = −0.41; *p* ≤ 0.05 for the autumn round, spring round and entire match season, respectively), ALT (R = −0.33; *p* ≤ 0.05, R = −0.40; p ≤ 0.05, and R = −0.4; *p* ≤ 0.05 for the autumn round, spring round and entire match season, respectively), CK (R = −0.22; *p* ≤ 0.05, and R = −0.23; *p* ≤ 0.05 for the autumn and spring round, respectively), LDH (R = −0.26; *p* ≤ 0.05, R = −0.31; *p* ≤ 0.05, and R = −0.22; *p* ≤ 0.05 for the autumn round, spring round and entire match season, respectively), ALP (R = −0.30; *p* ≤ 0.05, R = −0.60; *p* ≤ 0.05, and R = −0.39; *p* ≤ 0.05 for the autumn round, spring round and entire match season, respectively) and the level of urea (R = −0.20; *p* ≤ 0.05, R = −0.26; *p* ≤ 0.05, and R = −0.33; *p* ≤ 0.05 for the autumn round, spring round and entire match season, respectively). Only in the case of the creatinine level was a positive correlation in this group noted (R = 0.26; *p* ≤ 0.05).

When the midfielders and defenders were analyzed as individual subgroups, a significant negative correlation between the activities of AST and the cumulative game time was observed in both subgroups (R = −0.50 (Figure 10A); *p* ≤ 0.05, and R = −0.32; *p* ≤ 0.05 in the midfielders during the autumn and spring round, respectively, and R = −0.37; *p* ≤ 0.05 (Figure 10B), and R = −0.33; *p* ≤ 0.05 in the defenders during the autumn and spring round, respectively). The ALT was slightly negatively correlated with time only in the midfielders (R = −0.25; *p* ≤ 0.05 in the autumn round). In both subgroups, the activity of creatine kinase significantly increased with time during the entire season (R = 0.30; *p* ≤ 0.05, and R = 0.23; *p* ≤ 0.05 in the midfielders and defenders, respectively). In turn, the activity of alkaline phosphatase increased with time in the autumn round and decreased in the spring round (R = 0.18; *p* ≤ 0.05, and R = −0.39; *p* ≤ 0.05 in the midfielders during the autumn and spring round, respectively, and R = 0.29; *p* ≤ 0.05, and R = −0.35; *p* ≤ 0.05 in the defenders during the autumn and spring round, respectively). Additionally, in players from both positions, a negative correlation between the game time and the magnesium level was noted (R = −0.19; *p* ≤ 0.05, and R = −0.30; *p* ≤ 0.05 in the midfielders during the autumn and spring round, respectively, and R = −0.32; *p* ≤ 0.05 in the defenders only during the spring round). On the other hand, only in the midfielders was a decrease in the iron level (R = −0.33; *p* ≤ 0.05 in the entire season) and the LDH activity (R = −0.33; *p* ≤ 0.05 in the spring round) with time observed. The creatinine level increased significantly with time, especially in the autumn round in this group (R = 0.41; *p* ≤ 0.05, and R = 0.17; *p* ≤ 0.05 in the autumn round and entire season, respectively). In turn, in the defender subgroup, the LDH activity decreased with time in the spring round but increased in the total season (R = −0.29; *p* ≤ 0.05, and R = −0.23; *p* ≤ 0.05 in the midfielders during the spring round and entire season, respectively). Contrary to the midfielders, a negative correlation between the creatinine level and the cumulative game time (R = −0.28; *p* ≤ 0.05) and a positive correlation in the case of the iron level in the defender group (R = 0.23; *p* ≤ 0.05) were observed.

## 4. Discussion

Nowadays, a range of recovery tools is available, which can reduce recovery time and make it more efficient [30]. Several markers are used to assess post-match fatigue in soccer players in order to ensure optimal physical performance in every match [6]. Considering the diversity of responses to physical effort among players, combining fatigue markers of different types (performance, cognitive, subjective and biochemical markers) is strongly recommended [22]. When a new player joins a soccer team in the middle of a season, the rest values of CK, which is the muscle damage marker, are not available for coaching staff. There is therefore a need for reliable, valid and inexpensive biochemical markers that could inform coaches about the appropriate form and course of recovery for professional and amateur athletes. Some researchers suggest that blood sampling should be regularly carried out, and a database should be built for each individual player [31]. The restoration of metabolic homeostasis, muscle damage and decreases in performance usually takes 48–72 h in soccer players [22], although several of muscle related parameters have been normalized 24 h after a match [23], which seems to be the appropriate time to assess the levels of recovery biomarkers. The routine blood parameters are usually screened in soccer clubs. Therefore, there is an opportunity to extend a panel of laboratory tests and to verify them as recovery markers.

Repeated standardized blood sampling has been carried out, after each match at the time of the recovery session, over an entire competitive season (eleven months) in elite professional soccer players. The fasting capillary blood sampling was conducted 44 times to determine the AST, ALT, CK, LDH, ALP, creatinine, urea, iron and magnesium levels in the athletes. This long-term observation permitted a reliable assessment of the influence of regular intense training and competition on routine blood parameters, which can serve as a recovery efficiency marker.

The results of this study indicated that the activities of both aminotransferases (AST and ALT) were higher in the midfielder/defender group (Table 3), especially in midfielders (Table 4), but only AST significantly varied with time in this group. AST decreased in the autumn round in the midfielder/defender group (Figure 1C) and in the spring round only in the midfielders’ position (Figure 1A). The analysis of the correlations showed a significant decrease of this muscle origin variable with time in all of the studied subgroups. This indicates that players adapt to increases in training. This study produced results that corroborate the findings of Andelković et al., where male soccer players were examined during half of a competitive season [21], and Alaphilippe et al., who surveyed young elite rugby players during a sport season [32]. In another paper, where young footballers were assessed, a reduction in the AST activity was also observed. However, this study was conducted during a rest period [33]. It is worth mentioning that our study approach differs from the cited surveys, and for the first time, the data from such a long period of time (eleven months) are presented. In turn, the studies of Huggins et al. showed that the levels of aminotransferases were elevated in male soccer players in comparison to pre-season measurements [34]. Nevertheless, this research cannot be easily compared with the present study because that of Huggins et al. had a much shorter duration (lasting four months), and the adaptation process may therefore be incomplete. By comparison, our study had a duration of eleven months. Many others studies, in which the aminotransferase activity levels, after physical effort, were assessed, pointed out that AST and ALT were significantly elevated [15,16,32,35,36,37]. Moreover, an increase of the activity of these enzymes in peripheral blood might appear earlier than an increase in the CK activity [16], which makes it valuable in recovery efficiency assessing. The comparison of aminotransferases activity between the autumn and the spring rounds showed a significantly lower level of AST in the spring round than in the autumn round in all studied field positions. On the other hand, the ALT activity in the spring was significantly higher in the midfielders and lower in the defenders. This variability may be dependent on the succession of the seasons. In the study of Miyake et al., when 1,267,000 individual results from hospital databases were analyzed, the AST and ALT activities showed seasonal variations with the highest values in the winter and the lowest in the summer [38]. When circannual rhythm was studied in soccer players, the seasonal fluctuations of hormones like cortisol (with the peak in the winter) and testosterone (with the peak in the summer) were noted [39], which may influence body metabolism. It was found that testosterone independently correlates with the AST/ALT ratio [40] and cortisol stimulates aminotransferases [41]. However, in our study, the AST activity consistently decreased over the entire period of observation. Moreover, the higher levels of the ALT activity in the spring, only in the most active position of midfielders, were noted. It, therefore, appears that the annual changes of the aminotransferases activities noted in our group are mostly the result of adaptation to increases in training. For that reason, AST seems to be an appropriate biomarker of recovery efficiency, which is not significantly affected by other factors.

In the current study, none of the subgroups significantly differed in the creatine kinase activity levels. This is likely due to the large interindividual variability in the CK activities. Nonetheless, it was noticed that the CK activity varied significantly with time in the midfielder/defender group (particularly in midfielders) during the spring round of the soccer season. It can also be seen that a weak positive significant correlation between the CK level and the cumulative match-time was present in this group. The most active players, like midfielders, perform immense physical effort during each soccer match, so it is therefore not surprising that an increasing trend in the CK level was observed in this subgroup. These results are consistent with the work of other researchers, where the creatine kinase level was also found to have increased [6,31,34,42,43,44]. In fact, the soccer players are expected to have permanently elevated CK values, because they are exposed to training or match effort every day [3,45]. Moreover, the study of Souglis et al. confirmed the different responses over time between playing positions, where significantly, the highest peaks in the CK levels were observed in the midfielders compared with the defenders [46]. One of the reasons for changing the CK activity during the annual observation might be seasonal fluctuations. The recently published rhythmometric analyses of psychophysical markers in soccer players did not reveal the presence of a significant circannual rhythm in the CK activity [39]. Therefore, this factor is excluded as a cause of the CK activity increase. However, considering that a large interindividual variability of this muscular origin enzyme exists, CK seems to be an unsuitable parameter for monitoring the recovery process.

The current study found that the lactate dehydrogenase activity and urea level were surprisingly the highest in the goalie/substitute group, compared with the midfielder/defender group, and a decreasing trend of the levels of these variables over time was noticed in these groups (a significant negative correlation of LDH and urea with match-time for the season).

A number of studies have shown a significant post-effort increase in the LDH activity, for example, after rugby and soccer matches [29,47,48] or short-term maximal exercise (Wingate test) [49]. The differences between the subgroups in this study in terms of the LDH activity might be the result of the poor adaptation of the goalie/substitute group, caused by the significant differences in the time spent on the pitch or the extent of physical effort during the match. Another possible explanation for this observation might be the fact that the LDH activity could also depend on diet, which makes this parameter less effective [50,51]. In the current study, lactate dehydrogenase significantly varied over time in the midfielder/defender group for the whole season and also in the goalie/substitute group during the autumn round. The analysis of the correlations showed that the LDH activity slightly decreased with time in the midfielder/defender group (spring round) and in the goalie/substitute group (whole season). However, the findings of the current study do not support all of the previous studies. In the survey of Huggins et al., the LDH activity in soccer players remained unchanged for twelve weeks of the collegiate season [34]. On the other hand, when the LDH activity level in the competitive soccer half-season was measured, a significant decrease in the plasma activity of this enzyme was noted [21].

In the current study, the higher levels of urea seen in the players who spent much less time on the pitch or were less active (goalkeepers and substitutes), might imply a less efficient physiological adaptation of their organisms. It appears that even the best professional trainings do not replace the active participation in matches and competitions. Manna et al. suggested that the measurement of the urea level might be used to assess the training load of the players, because this parameter was independent of the age in their study. Moreover, the researchers postulated the use of urea as an index of adaptation to training. A low concentration of urea indicates the need for an increase in the levels of the exercise load [11]. On the other hand, unless the midfielder/defender group had a lower urea level than the goalie/substitute group, the statistical analyses showed that there was a slight but significant increase of this parameter with time in the spring round. Moreover, the midfielders displayed a higher blood urea level in the spring round when compared to the autumn round. It seems that regular players are characterized by a more efficient energy metabolism, where the protein breakdown for gluconeogenesis is lower, but the situation slightly deteriorates at the end of the spring round, which is the final round, and the players are prone to overreaching. It seems to be independent of a circannual rhythm of the cortisol level with the peak in the winter [39], which influences the aminoacid metabolism and the urea level [41].Some studies showed an initial increase and subsequent stabilization of the urea level during the competitive phase of the season [11,52].Others did not support these findings. By contrast, no changes in the blood urea concentration over time in the soccer players monitored during the season was found in the studies of Alaphilippe et al., Andelcović et al. and Huggins et al. [21,32,34]. A stable post-match urea level was also noted in the research of Colombini et al., suggesting that a low value of this variable is a recovery marker [53]. Using urea as an indicator for assessing the degree of body fatigue and the recovery process remains a debatable issue.

Creatinine is a metabolic product of a creatine breakdown. Prior studies have noted that athletes and active people, with a higher muscle mass (higher BMI), also have higher serum creatinine levels. This is the result of an increased creatine turnover [7,53]. The current study found that the Cr concentration was higher in the midfielder/defender group (particularly in midfielders) than in the goalie/substitute group. The non-parametric analysis of variance showed significant increases within the midfielder/defender group (entire season) and also in the goalie/substitute group (autumn round). Moreover, a significant positive correlation of this variable with the cumulative match-time was observed in the midfielder/defender group (especially in midfielders) and in the goalie/substitute group (autumn round only). These results differ from the study published by Alaphilippe and colleagues which showed no variation of the Cr level over time [32]. A small change or no change in the serum Cr concentrations in soccer players [32,42] posed a challenge to the frequent Cr level monitoring of elite soccer players [52]. On the other hand, our results are consistent with some other surveys. The significant changes in the Cr level over a three-month period of monitoring biomarkers in soccer players were found [21,34]. An increase in the creatinine post-match level, in comparison with the pre-match values, was also noted [53]. This is another reason in support of monitoring creatinine throughout the season.

The results from our study indicate that there were no significant differences between the midfielder/defender and the goalie/substitute groups in the alkaline phosphatase activity levels. However, the ALP activity changes with time in the midfielder/defender group during the autumn round were found. The analyses of the correlations showed an increase in the ALP activity with time in the autumn round and a subsequent decrease in the spring round, which might be the effect of adaptation. Nonetheless, these results differ from some published studies [34,54], in which neither the variation over time, nor post-effort changes, were observed. It was also proposed that measuring the post-effort ALP activity could be used in the detection of early symptoms of vitamin B6 and niacin deficiency in athletes [54,55]. In the current study, the total activity of ALP was measured, not only the activity of bone ALP, which was found to correlate with the duration of activity in male soccer players [56]. This seems to be a limitation of our study.

The current study noted a significantly lower level of iron in the midfielder/defender group, compared to the goalie/substitute group. Moreover, a decrease in the iron level with time in the spring round was observed. Additionally, the analysis of the correlations showed a slight decreasing trend in the iron level with time in the midfielder/defender group (R= −0,15), especially in midfielders (R = −0,33), for the whole season. The midfielders showed also a lower iron plasma level in the spring round, when compared to the autumn round. On the other hand, the results of the hematological tests did not reveal any aberrant values (data not presented). It is common knowledge that midfielders cover the longest distances during the match, in comparison with the players of other tactical formations [57,58]. It is therefore likely that such fluctuations in the iron level are the result of physiological stress, induced by prolonged and more intensive physical activity of this study group throughout the season. This finding is in agreement with Jamurtas’s [59] findings, which showed that match effort only transiently affects iron metabolism. In reviewing the literature, no iron deficiency in soccer players was found by many other researchers [21,34,42,60]. However, sometimes soccer players also have a slightly lower level of ferritin throughout the competitive season, which has very little or no influence on the players’ performance, but the iron status should be screened for health reasons [31].

Unless there were no significant differences in the magnesium levels between the subgroups, a negative correlation between magnesium and the cumulative match time in the midfielder/defender group was observed. In turn, the midfielders displayed a higher plasma magnesium level in the spring round, when compared to the autumn round. A significant variation of the Mg level in soccer players was also observed by Huggins et al. [34]. Since Mg has a role in glucose availability in the peripheral and central nervous systems and increases lactate clearance in muscles during exercise [61], particular attention should be paid to magnesium supplementation in elite players who are the most active on the pitch during the season.

It should be noted that, in the goalie/substitute group, a growing body of studied biomarkers were negatively correlated with the cumulative match-time. It seems that this may be the effect of adaptation to physical stress in this group, which had a much lower physical stress load than players from the midfielder and defender positions.

## 5. Conclusions

A long-term measurement of biochemical parameters in elite soccer players indicated that AST, CK, LDH and creatinine levels, when analyzed together, could constitute a useful set of markers in monitoring the recovery period of athletes. AST, LDH and Cr seem to be particularly good indicators because of the lower interindividual variability between these parameters in comparison to CK. 

The use of this combination of different routine blood biomarkers might tell more about recovery efficiency, augment positive adaptation and diminish the risk of overreaching or injury in soccer players. Moreover, it is inexpensive, fast and easy to run, and the panel of biochemical parameters is measured in capillary blood, not in venous blood, which seems to be another advantage.

Special attention should be paid to those players who have positions on the field which are more demanding, like midfielders, because they are more prone to deficiencies in ions, such as iron and magnesium, especially in spring.

## Figures and Tables

**Table 1:** A summary of the players’ game time.

Competitive Round	Blood Draws	Week’s Match-Time (min)	Cumulative Match-Time (min)
	baseline	0	0
**Autumn round**	1st	90 (82–90)	90 (60–90)
	2nd	58 (32–81)	137 (76–170)
	3th	89 (6–90)	206 (80–251)
	4th	90 (22–90)	234 (80–339)
	5th	90 (53–90)	283 (86–420)
	6th	90 (81–90)	322 (118–480)
	7th	82 (45–90)	377 (138–502)
	8th	90 (54–90)	457 (138–564)
	9th	90 (17–90)	502 (138–655)
	10th	80 (45–90)	542 (138–710)
	11th	60 (25–90)	561 (138–721)
	12th	90 (81–90)	628 (167–811)
	13th	90 (78–90)	675 (214–900)
	14th	31 (21–90)	695 (215–931)
	15th	90 (84–90)	724 (215–991)
	16th	90 (84–90)	730 (245–1046)
	17th	90 (45–90)	820 (275–1068)
	18th	34 (0–44)	830 (275–1090)
	19th	90 (82–90)	865 (275–1180)
	20th	90 (65–90)	907 (285–1270)
	21st	90 (74–90)	1047 (330–1367)
	22nd	90 (80–90)	1076 (339–1399)
	23th	90 (74–90)	1109 (384–1444)
**Spring round**	24th	90 (80–90)	1204(426–1572)
	25th	90 (88–90)	1293 (438–1576)
	26th	90 (89–90)	1338 (426–1661)
	27th	90 (82–90)	1348 (429–1751)
	28th	90 (8– 90)	1364 (433–1777)
	29th	90 (64–90)	1412 (433–1800)
	30th	90 (64–90)	1453 (464–1890)
	31st	90 (60–90)	1484 (464–1935)
	32nd	90 (0–90)	1529 (464–1980)
	33th	90 (35–90)	1570 (499–2070)
	34th	90 (0–90)	1619(330–2071)
	35th	90 (64–90)	1685 (536–2114)
	36th	90 (85–90)	1741 (562–2204)
	37th	90 (88–90)	1802 (607–2276)
	38th	90 (85–90)	1866 (652–2334)
	39th	90 (71–90)	1911 (697–2424)
	40th	90 (85–90)	2001 (747–2514)
	41st	90 (51–90)	2026 (792–2545)
	42nd	90 (0–90)	2036 (792–2565)
	43th	25 (45–90)	2041 (882–2575)
	44th	90 (61–90)	2104 (923–2608)

Data are presented as the medians (interquartile ranges).

**Table 2:** Baseline characteristics of the participants.

Parameter	Midfielders	Defenders	Goalie/Substitute
n	7	7	6
Age (years)	25 (21–30) *	26 (21–30) *	21 (19–25) *
Height (cm)	179.0 (175–187)	182.0 (181–185)	188.5 (188–190)
Weight (kg)	70.6 (68.3–80.7)	77.0 (73.0–80.0)	82 (78–87)
Length of training experience (years)	16 (15–19)	15 (12–18)	14.5 (9.0–16.0)
The number of weekly training hours (h)	13 (12–17.5)	12 (10–17.5)	12 (11–15)
Body Mass Index (kg/m^2^)	22.4 (21.6–24.3)	22.9 (22.5–24.2)	23.1 (21.7–24.4)
Entire season match-time (min)	2492 (1304–2680)	2156 (989–2643)	487.5 (366–2042)

Data are presented as the medians (interquartile ranges), n—number of participants, * median (minimum–maximum).

**Table 3:** Comparison of the midfielder/defender and goalie/substitute subgroups in terms of selected plasma biomarker values during the soccer match season.

	Entire Season			Autumn Round			Spring Round		
	Midfielder/Defender	Goalie/Substitute	*p*	Midfielder/Defender	Goalie/Substitute	*p*	Midfielder/Defender	Goalie/Substitute	*p*
**AST** (U/L)	39.7 (33.4–47.7)	36.15 (27.2–50.7)	0.007 *	42.0 (35.9–51.1)	40.9 (30.3–58.0)	0.753	36.6 (30.3–43.4)	31.0 (24.9–42.4)	0.003 *
**ALT** (U/L)	19.4 (14.6–25.1)	16.6 (11.9–26.8)	0.026 *	19.6 (16.0–25.1)	17.7 (125–28.9)	0.901	19.3 (14.4–25.0)	15.5 (11.9–24.1)	0.011 *
**CK** (U/L)	177.6 (106.1–315.4)	212.3 (85.9–454.8)	0.175	161.0 (91.5–288.8)	187.0 (73.6–479.9)	0.546	205.6 (123.4–352.2)	268.8 (114.4–454.6)	0.314
**LDH** (U/L)	314.0 (233.0–386.0)	348.8 (269.0–445.5)	0.001 *	296.0 (236.0–361.0)	354.0 (264.0–443)	0.001 *	336.0 (196.0–401.0)	358.0 (291.0–448.0)	0.034 *
**ALP** (U/L)	78.3 (62.7–94.9)	77.2 (45.5–95.3)	0.417	80.9 (68.9–93.6)	77.8 (54.2–91.5)	0.250	76.0 (20.6–97.7)	70.0 (50.16–101.75)	0.201
**Cr** (mmol/L)	107.0 (99.0–115.0)	102.5 (96.5–109.0)	0.001 *	109.0 (98.5–116.0)	104.0 (98.0–1100)	0.016 *	106.0 (99.0–113.0)	100.0 (95.0–108.0)	0.001 *
**U** (mmol/L)	5.4 (4.5–6.3)	5.7 (4.9–6.6)	0.006 *	5.4 (4.6–6.5)	5.9 (5.1–6.8)	0.003 *	5.4 (4.5–6.2)	5.4 (4.8–6.4)	0.341
**Mg** (mmol/L)	0.78 (0.73–0.83)	0.79 (0.74–0.83)	0.133	0.78 (0.73–0.83)	0.79 (0.75–0.83)	0.086	0.78 (0.72–0.83)	0.78 (0.73–0.83)	0.583
**Fe** (µmol/L)	16.1 (10.8–21.5)	17.4 (12.7–22.8)	0.004 *	17.3 (12.1–22.8)	17.8 (14.2–23.3)	0.188	13.8 (8.5–19.9)	16.6 (12.0–22.5)	0.003 *

Data are presented as the medians (interquartile ranges). Plasma biomarker values were compared using a Mann-Whitney U test. * Statistically significant difference; AST—aspartate aminotransferase; ALT—alanine aminotransferase; CK—creatine kinase; LDH—lactate dehydrogenase; ALP—alkaline phosphatase; Cr—creatinine; U—urea; Mg—magnesium; Fe—iron.

**Table 4:** Comparison of the midfielder and defender subgroups in terms of selected plasma biomarker values during the soccer match season.

	Entire Season			Autumn Round			Spring Round		
	Midfielder	Defender	*p*	Midfielder	Defender	*p*	Midfielder	Defender	*p*
**AST** (U/L)	42.0 (35.4–49.4)	39.0 (33.0–46.9)	0.010 *	43.4 (38.2–51.8)	41.7 (35.8–50.3)	0.144	39.6 (32.8–46.7)	35.4 (29.2–43.4)	0.023 *
**ALT** (U/L)	21.3 (17.1–29.1)	19.6 (14.6–24.1)	0.001 *	19.7 (16.1–26.2)	21.8 (18.4–26.3)	0.399	23.9 (17.5–31.9)	18.2 (13.1–22.6)	0.001 *
**CK** (U/L)	174.8 (110.7–307.4)	204.3 (100.5–358.4)	0.392	145.0 (89.1–254.8)	179.4 (79.8–331.4)	0.343	206.8 (138.2–332.1)	237.2 (109.3–380.8)	0.827
**LDH** (U/L)	309.5 (228.0–379.0)	322.0 (236.0–403.0)	0.179	288.0 (233.0–346.0)	300.0 (241.0–372.5)	0.150	325.0 (223.0–397.0)	349.0 (188.0–428.0)	0.412
**ALP** (U/L)	78.1 (60.0–93.6)	76.53 (63.3–90.3)	0.645	82.0 (65.1–94.8)	76.89 (68.6–85.7)	0.481	76.1 (22.9–95.1)	74.6 (19.3–101.9)	0.849
**Cr** (mmol/L)	107.0 (99.0–112.0)	106.0 (93.0–115.0)	0.392	107.0 (97.0–114.0)	107.0 (96.0–16.0)	0.719	106.0 (100.0–111.0)	104.0 (92.0–114.0)	0.827
**U** (mmol/L)	5.4 (4.6–6.0)	5.4 (4.4–6.6)	0.595	5.1 (4.5–5.9)	5.7 (4.5–8.8)	0.029 *	5.5 (4.8–6.2)	5.2 (4.3–6.3)	0.109
**Mg** (mmol/L)	0.78 (0.73–0.83)	0.78 (0.72–0.83)	0.754	0.77 (0.74–0.82)	0.78 (0.73–0.83)	0.586	0.79 (0.73–0.83)	0.77 (0.71–0.84)	0.379
**Fe** (µmol/L)	16.1 (11.0–22.5)	15.6 (9.5–20.9)	0.140	17.0 (12.9–24.8)	17.3 (11.4–22.0)	0.280	14.1 (8.4–20.2)	12.6 (7.9–19.1)	0.292

Data are presented as the medians (interquartile ranges). Plasma biomarker values were compared using a Mann-Whitney U test. * Statistically significant difference; AST—aspartate aminotransferase; ALT—alanine aminotransferase; CK—creatine kinase; LDH—lactate dehydrogenase; ALP—alkaline phosphatase; Cr—creatinine; U—urea; Mg—magnesium; Fe—iron.

**Table 5:** Comparison of the midfielder, defender and goalie/substitute subgroups in terms of selected plasma biomarkers values during the autumn and spring match rounds.

	Midfielder			Defender			Goalie/Substitute		
	Autumn Round	Spring Round	*p*	Autumn Round	Spring Round	*p*	Autumn Round	Spring Round	*p*
**AST** (U/L)	43.4 (38.2–51.8)	39.6 (32.8–46.7)	0.0003 *	41.7 (35.8–50.3)	35.4 (29.2–43.4)	0.0000 *	40.9 (30.3–58.0)	31.0 (24.9–42.4)	0.0000 *
**ALT** (U/L)	19.7 (16.1–26.2)	23.9 (17.5–31.9)	0.009 *	21.8 (18.4–26.3)	18.2 (13.1–22.6)	0.0005 *	17.7 (125–28.9)	15.5 (11.9–24.1)	0.1688
**CK** (U/L)	145.0 (89.1–254.8)	206.8 (138.2–332.1)	0.0002 *	179.4 (79.8–331.4)	237.2 (109.3–380.8)	0.0743	187.0 (73.6–479.9)	268.8 (114.4–454.6)	0.4669
**LDH** (U/L)	288.0 (233.0–346.0)	325.0 (223.0–397.0)	0.1156	300.0 (241.0–372.5)	349.0 (188.0–428.0)	0.6596	354.0 (264.0–443)	358.0 (291.0–448.0)	0.4246
**ALP** **(U/L)**	82.0 (65.1–94.8)	76.1 (22.9–95.1)	0.8662	76.89 (68.6–85.7)	74.6 (19.3–101.9)	0.0675	77.8 (54.2–91.5)	70.0 (50.16–101.75)	0.7366
**Cr** (mmol/L)	107.0 (97.0–114.0)	106.0 (100.0–111.0)	0.4758	107.0 (96.0–116.0)	104.0 (92.0–114.0)	0.1761	104.0 (98.0–1100)	100.0 (95.0–108.0)	0.0745
**U** (mmol/L)	5.1 (4.5–5.9)	5.5 (4.8–6.2)	0.03 *	5.7 (4.5–8.8)	5.2 (4.3–6.3)	0.0514	5.9 (5.1–6.8)	5.4 (4.8–6.4)	0.2619
**Mg** (mmol/L)	0.77 (0.74–0.82)	0.79 (0.73–0.83)	0.004 *	0.78 (0.73–0.83)	0.77 (0.71–0.84)	0.6596	0.79 (0.75–0.83)	0.78 (0.73–0.83)	0.4689
**Fe** (µmol/L)	17.0 (12.9–24.8)	14.1 (8.4–20.2)	0.0002 *	17.3 (11.4–22.0)	12.6 (7.9–19.1)	0.0049*	17.8 (14.2–23.3)	16.6 (12.0–22.5)	0.1554

Data are presented as the medians (interquartile ranges). Plasma biomarker values were compared using a Mann-Whitney U test. * Statistically significant difference; AST—aspartate aminotransferase; ALT—alanine aminotransferase; CK—creatine kinase; LDH—lactate dehydrogenase; ALP—alkaline phosphatase; Cr—creatinine; U—urea; Mg—magnesium; Fe—iron.
